# Diseased Antrum

**Published:** 1861-10

**Authors:** 


### DISEASED ANTRUM.

Mr. M—, with diseased antrum, whose case is described in the January number of the Register, p. 62, called for treatment again, September 18th, almost one year from the time the operation was first performed; the disease has continued, though with some abatement to the present time. The perforation into the antrum was kept open until within about one month, by wearing a wooden stopple in it. The second molar was crowded very much forward by the irruption of the wisdom tooth; by this means and persistent granulation the opening was closed; the discharge from the antrum was through the nostril; the discharge was a thick purulent matter and exceedingly fetid; it was far less in amount than a year ago, thicker and about the same in fetor.

We deemed it necessary again to make an opening into the antrum, and at the same point as before. A piece of gum some three lines in diameter was dissected between the bicuspid and second molar, then with medium sized trocar pierced the floor of the antrum, there was little or no discharge of pus, then syringed out effectually with warm water and tincture of arnica, directed gutta percha to be used as a stopple instead of wood as before.

At the suggestion of Dr. W. H. Mussey, I prescribed simple tincture of benzoin as a wash with which to inject the cavity, and five grains of iodide of potassa to be taken internally three times a day. Requested the patient to pursue that course which would procure the best passible state of health.

The general health of the patient is much better than it was a year ago, and upon this fact doubtless, the improved state of the antrum very much depends. He has been in camp about two months, and thinks his improvement was much more rapid than before.

This is one of the most obstinate cases of disease of the antrum with which we have met; there seems to be no cachectic condition upon which it can depend. The general health was good previous to its occurrence, and was only enfeebled by and in correspondence with this affection, at least this *seemed* to be the case; it is much less painful now than a year ago. We shall record from time to time the condition and treatment. T.

